# Altered Ca^2+^ Homeostasis in Immune Cells during Aging: Role of Ion Channels

**DOI:** 10.3390/ijms22010110

**Published:** 2020-12-24

**Authors:** Dorina Zöphel, Chantal Hof, Annette Lis

**Affiliations:** Center for Integrative Physiology and Molecular Medicine, Department of Biophysics, School of Medicine, Saarland University, 66421 Homburg, Germany; dorina.zoephel@uks.eu (D.Z.); s8cchoff@stud.uni-saarland.de (C.H.)

**Keywords:** calcium homeostasis, aging, T cells, T cell function, calcium, STIM, Orai, TRP channels, potassium channels, voltage-gated calcium channels, purinergic receptors

## Abstract

Aging is an unstoppable process and begins shortly after birth. Each cell of the organism is affected by the irreversible process, not only with equal density but also at varying ages and with different speed. Therefore, aging can also be understood as an adaptation to a continually changing cellular environment. One of these very prominent changes in age affects Ca^2+^ signaling. Especially immune cells highly rely on Ca^2+^-dependent processes and a strictly regulated Ca^2+^ homeostasis. The intricate patterns of impaired immune cell function may represent a deficit or compensatory mechanisms. Besides, altered immune function through Ca^2+^ signaling can profoundly affect the development of age-related disease. This review attempts to summarize changes in Ca^2+^ signaling due to channels and receptors in T cells and beyond in the context of aging.

## 1. Introduction

Aging is often associated with a loss of function. It describes a cumulative phenomenon that contributes to morbidity and mortality in man due to the greater incidence of infection, autoimmune phenomena, ineffective vaccination and cancer in elderly individuals (reviewed in [1,2,3,4,5]). One may also look at aging to be a constant adaptation and remodeling to the various, and often continuous, stressors encountered during life to maintain the organism’s overall functionality. The adaptation of cells during aging to environmental changes may increase the susceptibility to diseases but often ensures survival.

Dysregulation and changes of ionic fluxes across membranes mediated by ion channels, transporters, and receptors probably form the basis for a modified cell function not only during aging but also in disease. Ion channels and transporters evolved various mechanisms through which the monovalent (K^+^, Na^+^, Cl^−^) and divalent (Mg^2+^, Ca^2+^, Zn^2+^) ions are gated in response to different cellular signals.

Considering T cells, calcium (Ca^2+^) is one of the critical ions in generation, coordination, and control of signals within and between cells. To fulfill these numerous and wide-ranging tasks, very strictly coordinated and regulated calcium homeostasis and its maintenance in the cell is required. In the last decades of research, an incline of functional and signaling defects in elderly T cells has accumulated (reviewed in [6,7,8]). Most of these events depend either on transient or sustained Ca^2+^-influx to keep the intracellular calcium concentration [Ca^2+^]_i_ higher than basal levels for minutes to several hours. T cell function and maintenance are among the most remarkable and most pronounced changes occurring within an aging immune system.

Since the importance of Ca^2+^ for T cell function and in adaptive immunity has been already excessively and excellently reviewed by many groups [9,10,11,12,13], we focus more on the possibly altered channels and receptors during aging. These are expressed and play an essential role in T cells from human and mice. Although the signaling machinery in T cells is exceptionally complicated and many steps remain to be clarified, age-related changes in Ca^2+^ entry may be a critical cause of cell-mediated immune response decline with aging. This review reports findings on cellular mechanisms linked to Ca^2+^ homeostasis focusing on channels and their relevance in pathophysiological processes, mainly in T cells during aging.

## 2. Altered T Cell Function during Aging

One significant hallmark in immune system aging is the thymic involution with age, resulting in a steady decline of naïve T cells (T_N_) numbers [14,15,16,17] with restricted T cell receptor (TCR) repertoires [18,19,20], ending in disrupted T cell homeostasis. The lack of naïve T cells and increasing memory T cells (T_M_) accumulation contribute to a higher risk of severe infections in the elderly [21]. Interestingly, the naïve CD8^+^ T cell compartment is much more affected than the CD4^+^ T cells, including higher contraction rates [17,18,19]. However, a quantitative decrease of T_N_ cells alone might not account for the functional differences in effector (T_E_) and T_M_ cell responses. Different models are discussed in this context, such as distinctive phenotype between adult and elderly naïve CD8^+^ T cells with altered survival, developmental pathways, and responses to infection [22,23,24]. On the other hand, an increased adaptation to environmental changes during aging has been observed [25] consistent with loss of stem-like features lead to reduced plasticity [24,26] and accumulation of virtual-memory T cells without former antigen stimulation [27,28]. 

TCR antigen-binding triggers intracellular Ca^2+^ mobilization and is required for plethora of cellular processes and may account for reduced effector responses by aged T cells. Activation of TCR includes a firmly defined sequence of events, and numerous age-related deficits are described in T cell signaling pathways after its activation. Just recently, microRNA (miR-181a) expression emerged as a crucial regulator of controlling TCR activation thresholds in peripheral T cell response [29]. In naïve CD4^+^ T cells from elderly organisms, lower miR-181a expression leads to reduced extracellular regulated kinase (ERK) upon TCR activation [30], as well as in naïve CD8^+^ T cells [31]. The deletion of miR-181a in peripheral T cells in a mouse model causes defective viral response through impaired generation of CD8^+^ effector T cells [32]. Additionally, activation-induced upregulation of miR-21 shifts the transcriptome towards effector T cells and away from memory T cell differentiation [33]. 

Unfavorable alterations of T cells subpopulations result in a decreased CD4^+^/CD8^+^ ratio and the accumulation of senescent and terminally differentiated T cells (reviewed in [34,35]). The inversion of the CD4^+^/CD8^+^ ratio is associated with altered immune function, chronic viral infection, and chronic inflammation [36,37,38]. Besides the ratio, the CD4^+^ and CD8^+^ T cell subsets are affected differently by aging [39]. The aged naïve CD4^+^ T cells differentiate poorly to T-helper-cell-1 (Th1) and T-helper-cell-2 (Th2) effector subsets, but the ability to generate T-helper-cell-17 (Th17) is intact, reflected by their increased numbers during aging [40]. Moreover, the elderly have increased Th1/Th2 ratio [41], and data from murine studies supports a shift from a Th1-like to a Th2-like cytokine response [42]. Simultaneously, the subset of regulatory T cells (Tregs) increases compared to adult individuals [43]. The accumulation of functional Tregs contributes to the frequent reactivations of chronic infections often observed in aging. The aged-dependent decrease of Th17/Treg ratio after stimulation accompanying altered cytokine expression may contribute to the imbalance between pro-and anti-inflammatory immune responses [44]. 

Furthermore, in vitro stimulated T cells from humans and mice show altered cytokine secretion. However, the published results are ambiguous and inconsistent for many investigated cytokines, like interferon-gamma (IFN-γ) and interleukin-2 (IL-2) [40,41,45,46,47,48,49,50,51,52]. All the studies highlight the importance of used stimuli for cytokine induction and the resulting impact on immune responses. Additionally, one must consider T cell responsiveness’s altered kinetics with age as a possible cause impacting proliferation, upregulation of activation markers, and cytokine secretion [53]. Naïve CD4^+^ T cells from elderly mice secrete less than 50% IL-2 compared to adult cells, leading to decreased expression of CD25 (IL-2 receptor α), and show reduced proliferation and incomplete differentiation to effector cells [50,54]. The age-related reduction in IL-2 production by CD4^+^ T cells is not fully explained either by alterations of the actual structure of TCRs or by changes in the TCR-CD3 complex [55,56]. The defects in effector generation associated with aging are reversible by adding IL-2 but no other related gamma chain (γc)-receptor binding cytokines [50]. Already in 1985, the first experiments implicate a Ca^2+^ influx as an essential component for IL-2 function [57]. Nowadays, there is no doubt about the regulation of IL-2 and IL-2 receptor (IL2-R) mediated signaling through the nuclear factor of activated T cell (NFAT)/calcineurin pathway controlled by Ca^2+^ influx upon TCR and costimulatory signals [58,59].

The influence of aging is not only limited to T cell subtype distribution and cytokine production of CD4^+^ T cells but also the cytotoxicity of CD8^+^ T cells is changed. In a study by Fagnoni and colleagues, the CD3-mediated cytotoxicity of freshly isolated T cells from healthy aged donors against P815 target cells exhibited higher values than their younger counterparts [60]. This correlates with higher amounts of CD8^+^CD28^−^ cells in elderly humans [60,61]. However, in the context of disease, the elderly human with COVID-19 show reduced overall CD8^+^ T cell numbers and granzyme A expression by CD8^+^ T cells. In effector memory (T_EM_) and T_E_ cells, perforin’s expression decreases with age in those patients [62]. Furthermore, in vitro stimulated naϊve elderly CD4^+^ T cells exhibit impaired cytoskeleton signaling, LAT (linker of activated T cells) and ZAP-70 (Zeta-chain-associated protein kinase 70) recruitment, and CD3-zeta assembly with the cytoskeleton to the induction of NFAT [52,63]. Additionally, CD4^+^ T cells from aged TCR transgenic mice do not form immunological synapses (IS) with antigen-presenting cells (APC) as efficiently as in adult mice, with a reduction in the recruitment of signaling molecules in the elderly compared to adult CD4^+^ T cells [64,65]. 

Changes in Ca^2+^ influx in aged T cells are also reported [66,67]; however, the influence of aging after TCR activation and the underlying molecular players are still under investigation. Following T cell activation in mice, several groups reported a decline in the Ca^2+^ levels with age [68,69]. Comparing T cells from mice of any age, naïve T cells are much more likely than memory T cells to respond with an increase in [Ca^2+^]_i_ in response to lectin, anti-CD3 plus anti-CD28, or Ca^2+^ ionophores [70,71]. These studies suggest that naïve and memory T cells differ fundamentally in their ability to increase [Ca^2+^]_i_ following receptor-dependent or receptor-independent stimulation. Changes in basal Ca^2+^ levels reported by several studies are conflicting. The resting level of free Ca^2+^ is lower or unaffected in human aged T cells [72,73] but higher in T lymphocytes obtained from elderly mice [74]. 

Many of the dysregulations described above are highly dependent or regulated by Ca^2+^ itself; however, the underlying molecular mechanisms are not well characterized and still under investigation. Altered Ca^2+^ fluctuations have already been associated with numerous age-related diseases, such as neurodegenerative [75], muscle-related diseases [76,77], autoimmune and inflammatory disorders [78,79]. Ca^2+^ responses are regulated negatively and positively by several mechanisms involving channels, pumps, and sensors (reviewed in [80]). Here we review the impact of possibly altered Ca^2+^-permeable channels expressed in T cells and their contribution to the altered processes observed during aging. 

## 3. Orai/STIM

In many immunocytes, the main mechanism for Ca^2+^ entry is through SOCE (store-operated Ca^2+^ entry) [81] and involves the activation of CRAC (Ca^2+^-release activated Ca^2+^) channels. Genome-wide RNAi screens and linkage analysis in human patients with defects in SOCE identified two fundamental players of SOCE via I_CRAC_ [82]: Stromal interaction molecules (STIM1), as the ER Ca^2+^ sensor [83,84] and the CRAC channel [85,86,87] itself. In addition to the identification and characterization of the Orai and STIM homologs [88,89,90], the research of the last 15 years has revealed numerous splice variants [91,92,93] that contribute to the diversity of the resulting Ca^2+^ signals. 

SOCE pathway is important for the immunocytes and essential for numerous cellular processes, including sufficient T cell activation, development, differentiation, gene expression, the formation of the immunological synapse, and cytotoxicity (reviewed in [10,13,58,94,95,96]). For efficient development of an immune response, T cells require long-lasting Ca^2+^ influx through CRAC channels, and the formation of a stable IS with the antigen-presenting cell (APC) [97,98]. Orai1 and STIM1 translocate to IS accompanied by Ca^2+^ influx through CRAC channels [97,99,100]. Besides, mRNA expression for *STIM1* and *Orai* homologs is upregulated. The generated distinctive Ca^2+^ patterns determined by the heterogeneous composition of channels and activators [101,102,103] allow not only their modulation but the transmission of extracellularly generated signals intracellularly. 

The magnitude and duration of changes in [Ca^2+^]_i_ are crucial determinants for T cell activation and other immune system responses. Prolonged elevations of [Ca^2+^]_i_ are vital for activating transcription factors that initiate many changes in gene expression which drives T cell proliferation, cytokine, and chemokine production. The work in deficient mouse models gives an insight into the variety of processes mediated and determined by SOCE. Profound defects in key T cells cytokines such as IL-2, IL-4, IL-10, IFN-γ and TNF-α and apoptosis genes are found in CD4^+^ and CD8^+^ T cells from *Orai1*, *STIM1*, and *STIM1/2*-deficient mice [104,105,106]. Complete inhibition of SOCE in CD8^+^ T cells from *STIM1/2*-deficient mice impairs lytic granule exocytosis and elimination of tumor cells and virus-infected cells [107,108]. Additionally, CD8^+^ T cells and NK cells show Ca^2+^ dependent cytotoxicity with an optimum for cancer cell elimination at rather low free [Ca^2+^] concentrations. Downregulation of *ORAI1* in cytotoxic T lymphocytes (CTLs) leads to decreased Ca^2+^ signals but increased efficiency to eliminate cancer cells [109]. It seems like delineation of the accurate STIM/Orai ratio could be a feature of the killing efficiency of CD8^+^ T cells by determining the Ca^2+^ killing optimum.

One of the T cells’ best studied Ca^2+^-dependent mechanism is the NFAT (nuclear factor of activated T cells)/calcineurin pathway [110]. The NFAT-driven gene expression is highly dependent on sustained Ca^2+^-influx. The activation of calmodulin-dependent enzyme calcineurin by the rise in [Ca^2+^]_i_ levels leads to NFAT dephosphorylation followed by nucleus translocation. A decrease in [Ca^2+^]_i_ levels leads to the export of NFAT from the nucleus [111]. Undoubtedly, the relevance of SOCE highlights the fact that lymphocytes with defective SOCE are unable to mount an immune response, and patients with such defects develop SCID. Studies of Orai1 in SCID patients have further confirmed that CRAC channels are the primary pathway for Ca^2+^ entry in naïve T cells. An Arg91Trp mutation in *Orai1*, as a pore-forming subunit of CRAC channels, is responsible for abolishing Ca^2+^ influx in T cells from these SCID patients [112]. Meanwhile, numerous other mutations in *STIM* and *ORAI* were identified, leading to distinctive phenotypes in patients (reviewed in [113]). Unexpectedly, immunodeficient patients with loss-of-function or null mutations in *ORAI1* or *STIM1* that abolish TCR-mediated Ca^2+^ influx in T cells have normal CD4^+^ and CD8^+^ T cell numbers with a normal TCR Vß repertoire [114,115]. These data indicate that CRAC channels do not play a significant role in the thymic development and selection of T cells. The functional defect is not limited to T cells and affects SOCE in B cells and fibroblasts [116]. Homozygous mice lacking STIM1, STIM2, or Orai1 are embryonic lethal or die soon after birth [104,105]. *STIM1*-deficient T cells completely lack SOCE, I_CRAC_, and Ca^2+^-dependent cytokine expression [105], but the *STIM2*-deficient naïve T cells show normal SOCE and cytokine production. T cells from *Orai1*-null mice also display an evident impairment in all three functions [104,105]. Moreover, *Orai1/Orai2*-deficient mice are protected from autoimmunity and alloimmunity in graft-versus-host disease. The deletion of *Orai1/Orai2* in T cells abolishes SOCE leading to augmented T cell function and altered proliferation and cytokine production. Surprisingly, *Orai2*^−/−^ T cells exhibit increased SOCE without improving T cell function in vivo and in vitro [117]. Additionally, Orai2 shapes the Ca^2+^ signaling profile in human Tregs after thapsigargin or TCR- induced SOCE. The enhanced Ca^2+^ signals, compared to the conventional CD4^+^ T cells, correlate with the lower expression of Orai2 in these cells [118].

Despite the substantial literature on SOCE associated with T cell function, the changes in Ca^2+^ homeostasis components and age-related changes in Ca^2+^ entry are less well understood. We recently linked the aging-related reduction in Ca^2+^ signals to reductions of the primary critical players in the Ca^2+^ signaling pathway [66]. The reduced expression of STIM and Orai mRNA and proteins leads to reduced Ca^2+^ entry. The upregulation of the plasma membrane Ca^2+^ ATPases 4 (PMCA4) contributes to faster extrusion in CD8^+^ T cells isolated from aged mice. Furthermore, these cells show a less efficient TCR-induced [Ca^2+^]_i_ mobilization and increased insensitivity to Ca^2+^ fluctuations during cytotoxic activity [66]. 

## 4. TRP Channels

Next to STIM and Orai, other Ca^2+^ and ion channels, including TRP channels, are relevant for Ca^2+^ signaling. TRPV1 contributes to the TCR-induced Ca^2+^ entry in CD4^+^ T cells and is gated by phosphorylation depending on the lymphocyte-specific protein tyrosine kinase (LCK) [119]. Complete deletion of *Trpv1* using a mouse model showed impaired TCR signaling resulting from reduced Ca^2+^ flux [119]. Furthermore, CD4^+^ T cells presented defects in T cell activation and cytokine production [119], also confirmed by using TRPV1 antagonists in T cells isolated from murine spleen [120]. However, additional electrophysiological data is missing to underline that TRPV1 is activated downstream of TCR. Besides, an inhibition of the TRPA1 channel can inhibit the TRPV1 activity, thereby reducing the Ca^2+^ influx. This inhibition is caused by a direct heteromerization of the two channels and such mutual modeling has also been described for other channels combination such as Orai1 and Orai2 [117] or TRPM7 and TRPM6 [121,122]. 

TRPC3, 5 and 6 are involved in T cell Ca^2+^ signaling. The Ca^2+^ influx via TRPC3 modulates cell proliferation [123,124]. TRPC5 seems to be important in mediating Treg-influenced inhibition of T_E_ cells however the exact mechanism remains elusive [125]. The involvement of TRPC channels in T cells remains highly argumentative. More detailed and sophisticated studies (not only during aging) are necessary to address these issues. 

TRPM2 is another channel expressed by different cell types of the peripheral immune system, including lymphocytes [126] and monocytes [127], which is involved in immune cells function. It is stimulated by oxidative stress and specifically activated by intracellular ADP-Ribose. Second messenger molecules like cyclic ADP-ribose (cADPR) and nicotinic acid adenine dinucleotide phosphate (NAADP) can activate and regulate the Ca^2+^ influx through TRPM2 channels in lymphocytes [128,129]. TCR engagement causes a sustained cADPR increase and an antagonist of cADPR inhibits T cell activation and proliferation in response to T cell stimulation [129]. However, it needs to be considered that this effect can also be explained by the effect of cADPR on RyRs. *Trpm2*^−/−^ T cells exhibit reduced proliferation and proinflammatory cytokine secretion [130]. More evidence for the role of TRPM2 in inflammation can be found after induced inflammation by H_2_O_2_ or lipopolysaccharide (LPS). TRPM2 activation promotes immune responses through cytokines production like CXCL8, IL-6, IL-10 and TNF-alpha in monocytes [127,131]. The incubation of monocytes with LPS resulted in TRPM2 mRNA and protein upregulation and ADP-ribose-induced membrane currents [127]. By *Trpm2*-deficient mice, it was shown that TRPM2 minimizes excessive inflammation by dampening the inflammatory response through cellular depolarization and following reduction of ROS production in phagocytes [132]. The exposition to endotoxins demonstrated augmented inflammatory response and decreased survival compared to wild type mice. There is also good evidence that TRPM2 plays an essential role in ROS-coupled diseases since H_2_O_2_-mediated TRPM2 activation is a potential mechanism for pathogenic processes characterized by an increased oxidative microenvironment, including inflammation. Interestingly, its role in aging of immune cells has hardly been explored, although ROS plays a central role in aging theory (reviewed in [133]) and one could imagine TRPM2 physiological or pathophysiological role during age-associated inflammatory responses. The existing *Trpm2*-knock out mouse studies already provide initial information and coherences about this channel’s role in the development and course of the inflammatory processes because an exacerbating inflammation and age-related upregulation of pro-inflammatory cytokines were not observed in *Trpm2*-deficient mice at least in the brain [134]. However, more sophisticated studies are required to examine its role in immune cells in the aging context.

TRPM4 is involved in a diversity of physiological processes, including T cells [135], mast cells [136], and dendritic cells [137] activity. It has a profound impact on Ca^2+^ signaling because Na^+^ entry depolarizes the plasma membrane to reduce the driving force for calcium entry during SOCE. The Ca^2+^ induced TRPM4 activation serves as a negative feedback mechanism to prevent toxic Ca^2+^ overload and fine tunes T cell responses [135,138]. Small interfering RNA-mediated knockdown of *Trpm4* amplified Ca^2+^ entry, NFAT translocation, and IL-2 production in mouse Th2 cells, but it had the opposite effect in Th1 cells [139]. The reasons for the differences are the diverse TRPM4 expression levels as well as different Ca^2+^ clearance dynamics in subtypes, leading to reduced TCR-mediated Ca^2+^ influx in Th2 cells [139] and the high sensitivity to FAS-dependent apoptosis in Th1 cells [140]. The deletion of *Trpm4* gene impaired antigen- and stem cell factor- induced migration of bone marrow derived mast cells (BMMCs) [136] and chemokine-dependent migration of dendritic cells [137]. In a sepsis model, the ablation of *Trpm4* gene decreased phagocytic function and pro-inflammatory cytokine production leading to increased mouse mortality [141]. In BMMC’s, Ca^2+^ influx via CRAC channels decreases critically after TRPM4 channels depolarize the membrane following adenosine- and FcεRI-stimulation. Accordingly, activated *Trpm4*^−/−^ BMMCs have amplified degranulation and release excessive amounts of histamine, leukotrienes, and tumor necrosis factor [142]. Furthermore, TRPM4 channel activation is an efficient mechanism for limiting exaggerated antigen-induced mast cell activation that triggers inflammatory and allergic reactions [143]. 

The selective cation permeable channel TRPM7 with protein serine/threonine kinase activity [144,145,146] has been implicated in numerous physiological functions, including cell survival, proliferation, apoptosis as well as migration, and immune cell function (reviewed in [147,148,149,150,151,152,153]). TRPM7 is essential for T cell development since *Trpm7* knock out mice have reduced numbers of T cells due to halting of thymocytes development at the double negative CD4^−^CD8^−^ stage and resulted in altered chemokine and cytokine expression [154]. Moreover, the T cell specific *Trpm7* deletion in vivo resulted in reduced expression of essential growth factors and progressive loss of medullary thymic epithelial cells [154], which regulate T cell development through their function as APC. The impact on proliferation efficiency depends mainly on the type of activation stimuli [155,156]. The channel itself seems to regulate T cell homeostasis by mediating Fas-depending T cell apoptosis through caspase activation [157]. TRPM7 can activate SOCE by phosphorylation of CRAC components leading to reduced SOCE in the absence of TRPM7 [158] and is implicated in receptor-induced Ca^2+^ release [159]. The positive regulation of SOCE requires the channel kinase activity but not the channel domain itself. The inactivation of TRPM7 kinase activity by introducing the K1646R mutation shows reduced SOCE [155] but normal T cell development except for a reduction in Th17 cell development [156]. The fact that the Treg cells were not affected in this context was very thrilling since both originate from the same precursor cells, and their differentiation requires the involvement of the TGF-β (transforming growth factor ß) [160,161,162,163]. Overall, Th17 cells up-regulate inflammation, while Treg cells have an immunosuppressive function [164,165]. Altered balance of Th17/Treg may play a critical role in the pathogenesis of autoimmune and chronic inflammatory diseases (reviewed in [166]). To ensure an effective immune response, the inflammatory response must be tightly regulated to avoid damage and destruction. However, a characteristic feature of aging and aging-related disease is “inflamm-aging”, associated with immune imbalance and cytokine dysregulation (reviewed in [167,168,169,170]). Several studies already reported a reciprocal connection between pro-inflammatory Th17 and anti-inflammatory Treg cells [162,171] during aging [44,172]. The balance between Th17/Treg and their generation and maintenance are influenced by many factors including TCR and cytokine signaling [166]. The study of Romagnani and colleagues [156] implicated a distinctive defect of small mothers against decapentaplegic family member 2 (SMAD2) signaling in T cells and highlight the role of TRPM7 kinase inhibition in immune homeostasis and in graft-versus-host disease. Although current studies are missing, the existing data provides an excellent foundation to study the involvement of TRPM7 not only in the context of inflammation but also in aging.

## 5. Potassium Channels

After TCR activation in immune cells, subsequent opening of calcium-activated and voltage gated K^+^ channels (K_V_1.3, K_Ca_3.1) mediate K^+^ influx and hyperpolarization, providing an electrochemical gradient critical for sustained Ca^2+^ influx (reviewed in [173,174,175]). In T cells of human and mice several K^+^ channels have been reported and their expression depends on activation and differentiation status. Naïve human and mouse CD4^+^ and CD8^+^ T cells, as well as activated central memory T cells (T_CM_) predominantly express K_V_1.3 [176,177,178,179,180,181]. Furthermore, T cells from human and mice, upregulate the calcium-activated channel K_Ca_3.1 following T cell activation to maximize Ca^2+^ influx and proliferation during the re-activation of T_N_ and T_CM_ [177,178,182]. Additionally, the sensitivity to selective blockers of K_Ca_3.1 and K_V_1.3 differ in T_N_ versus T_M_ because of the different expression levels of these channels [176,177,178,180,183,184,185]. However, mouse T_EM_ up-regulate K_Ca_3.1 instead of K_V_1.3, like shown in humans and rats. Although in *K_Ca_3.1*-deficient mice the CD4^+^ T cell differentiation was not affected but Ca^2+^ influx and cytokine production in Th1 and Th2 cells were impaired in contrast to Treg and Th17 cells [182]. The results from the *K_Ca_3.1*^−/−^ mice underlie the role of K_Ca_3.1 function in the activation of CD4 subtypes [182].

Although the T cell homeostasis in humans and mice fundamentally differs [186], it is beyond question that these processes require stable and balanced calcium homeostasis [10]. A block of both K_V_1.3 and K_Ca_3.1 abolishes Ca^2+^ oscillations, impacting T cell proliferation [187]. Overall, the pharmacological inhibition of K^+^ channels reduces Ca^2+^ influx and decreases cytokine expression profile [182,188,189]. The discovery of immunomodulatory actions [190] by inhibiting K_V_1.3 channels pave the way for intensive investigations on a therapeutic application in immune-mediated disorders [177,180,191]. Besides, the differentiation of CD8^+^ T cells into effector cells with cytotoxic ability requires K_V_1.3 channels. K_V_1.3 channels gather specifically at the IS between cytotoxic and target cells to modulate the killing process mediated by cytotoxic T lymphocytes [192,193]. 

Changes in the prevalence of distinct T cell subsets have already been studied extensively [17,62,66,194], however very little is known on functional alterations affecting activation and the underlying molecular mechanisms (not only) in aging. The influence of T cell activation by Ca^2+^ influx regulated by K_V_1.3 and IK_Ca_1 potassium channels may alter T cell function during aging [195]. The use of specific inhibitors of K_V_1.3 and IK_Ca_1, namely margatoxin (MGTX) and triarylmethane-34 (TRAM), reveals a different pattern of Ca^2+^ influx kinetics dependent on age and T cell subset. High Ca^2+^ influx observed in CD8^+^, and Th1 T cells decreased during aging. Surprisingly, the Ca^2+^ influx in Th2 is similar in all investigated age groups. MGTX inhibitory effect is even more pronounced in Th2 cells, whereas in Th1, the TRAM inhibition remains more potent. Ca^2+^ influx of CD8^+^ T cells is inhibited to a similar extent by both applied inhibitors in the two adult groups and does not affect in the elderly. K_V_1.3 and IK_Ca_1 channel dysfunction, as essential regulators of Ca^2+^ influx kinetics, is associated with altered function and contribute to age-related changes of T cells [195]. Basically, any ion signaling dysregulation can have severe effects on immune function, leading to (age-related) diseases. During necrosis in the tumor microenvironment, exposure of T cells to high K^+^ concentrations inhibits T_E_ cell function. The excessive extracellular potassium concentration ([K^+^]_e_) leads to increased [K^+^]_i_ blocking the TCR/Akt/mTOR pathway via phosphatase [196]. The consequence is the inhibition of transcription of genes mediating T cells’ activation response to antigen presentation. 

## 6. CaV Channels, Voltage Gated Channels

T lymphocytes express, among others, the ß3, ß4, and α1 subunit of voltage gated channels Ca_V_1.1, 1.2, 1.3, 1.4, and Ca_V_3.1 [197,198,199,200,201]. The increasing number of publications conducted using mice models provided useful insights of Ca_V_1 channels and subunits in T cell biology and uncovered their role in the activation and survival of T cells [200,201,202,203]. Additionally, the plethora of newly discovered splice variants with altered gating characteristics [199] and partly complete insensitivity to membrane polarization [204] may play a critical role in shaping Cav-dependent Ca^2+^ signals [205]. Murine CD4^+^ and CD8^+^ T cells with a conventional *Ca_V_1.4*-deficiency showed impaired Ca^2+^ influx and decreased ERK (extracellular-signal-regulated kinase) and NFAT activation response to TCR stimulation [201]. Furthermore, *Ca_V_1.4*-deficiency is associated with increased apoptosis and a relative loss of naïve CD44^lo^ T cells in vivo. Upon infection, the number of functional Ag-specific T cells is reduced, shifting towards T_M_ cells phenotype with upregulated activation markers [201], and failed to mount an effective antigen-specific CD8^+^ T cell response. The lack of ß regulatory subunits in mice models resulted in compromised cytokine production in CD4^+^ T cells and decreased expression of the Ca_V_1.1 pore-forming units [199] required for TCR-induced Ca^2+^ entry [198]. The lack of ß3 subunit in CD8 T cells leads to reduced cell numbers due to spontaneous apoptosis mediated by high expression of the Fas receptor [200]. Like the CD4^+^ T cells, the remaining CD8^+^ T cells showed activated memory character and defects in TCR-induced Ca^2+^ signaling and proliferation. Moreover, the lack of ß3 subunit in naïve CD8^+^ T cells resulted in compromised Ca_V_1.4 protein expression, suggesting that Ca_V_1.4 and β3 may form a Ca^2+^ channel complex [200]. Ca_V_1.2 and Ca_V_1.3 may play a role in Th2 cell activation since their deletion impaired TCR-induced Ca^2+^ influx and IL-4 production in vitro and prevented experimental asthma development [202,206]. Finally, the T-type channel *Ca_V_3.1*-deficiency showed a protective role in EAE (Experimental Autoimmune Encephalomyelitis) mouse model due to reduced cytokine production of granulocyte macrophage colony-stimulating factor (GM-CSF), IL-17A, IL-17F, and IL-21 in Th1 and Th17 cells [197]. Although the overall published data implicate Ca_V_ channels’ involvement in Ag-receptor signaling, it is still a pending question how Ca_V_ channels work in T cells and in combination with other channels to shape a specific calcium signaling. Unfortunately, momentarily no data are available for Ca_V_ channels and function during aging in the immune system. The main reason might be the difficulty to separate the involvement of Ca_V_ in aged related defects in the interplay of channels, pumps, and receptors involved in the choreography of Ca^2+^ signaling in immune cells. 

## 7. Purinergic Receptors

The members of the P2X receptor family are widely expressed among human and mice immune cells. Probably, the best-studied and characterized, not only in T cells, is the P2X7 receptor with an established role in inflammatory and immune responses [207,208]. At this point, it is worthy of mentioning that besides the P2X5 receptor, all other family members can facilitate extracellular adenosine triphosphate (ATP)-mediated Ca^2+^ entry [209,210]. 

Upon TCR activation, the increase in mitochondrial activity requires an increase in cytosolic Ca^2+^ concentration via CRAC channels to raise ATP secretion leading to autocrine activation via P2X receptors. In turn, P2X receptor activation causes a Ca^+2^ influx, IL-2 production, and proliferation by the activation of NFAT along with an increased expression of the *P2RX7* gene [211,212]. ATP release via pannexin-1 hemichannels after TCR activation placed ATP as a mediator in an autocrine feedback loop intensifying T cell stimulation [213] and also helps to sustain P2 receptor signaling as well as NFAT activation [214]. A successful T cell activation requires forming of a stable IS with the antigen-presenting cell [215]. Pannexin-1 hemichannels, P2X1, and P2X4 receptors rapidly translocate to the IS after TCR stimulation facilitating ATP release and autocrine feedback mechanism, while P2X7 receptors remain uniformly distributed [216]. It implicates that P2X receptor subtypes may fulfill different functions in different steps during T cell activation. The colocalization with STIM1 and Orai1 enhances Ca^2+^ entry at the IS [216] which is necessary during weak TCR stimulation and supportive in antigen scanning or for the formation of Ca^2+^ microdomains. 

Besides the autocrine signaling during T cell activation, paracrine effects have been observed. Extracellular ATP, as a danger signaling molecule during inflammation and injury, activates the innate immune system and mediates chronic pain through P2X7 receptors [217,218]. However, the latest work showed the unsuspected but critical involvement of P2X7 in generating resilient, long-lived central and tissue-resident memory CD8^+^ T cells (CD62L^+^) supporting adaptive immune system memory [212]. The induction of adenosine monophosphate (AMP)-activated protein kinase, metabolic reprogramming and mitochondrial maintenance promotes T_CM_’s metabolic fitness while T_EM_ generation is much less affected [212]. On the other hand, P2X7 receptor inhibition supports CD4^+^ T cells’ differentiation into Tregs [219]. Furthermore, P2X7 and P2X4 receptors play an essential role in non-conventional γδ T cell differentiation and cytokine production mediated by amplification of TCR-mediated Ca^2+^ signaling [220,221]. Application of extracellular ATP on *P2RX7*-deficient T cells prevents shedding of CD62L (L-selectin) [222]. Furthermore, P2X7 receptor seems to be essential for ATP-induced shedding of CD23, CD27, and IL-6R mediated by metalloproteases and converts the membrane proteins into soluble effector proteins [222,223,224,225]. Additionally, ATP concentration seems to determine T cells’ fate, whether to keep them in a resting state, becomes activated, or undergo apoptosis [226]. In mature T cells, the P2X7 receptor is essential for the induction of apoptosis by ATP [227] and nicotinamide adenine dinucleotide (NAD) [228]. One another fascinating paracrine ATP-function is the influence of the P2X7 and X4 receptors on the migration or motility of T cells [229]. In lymph nodes, ATP-release from activated T cells reduces bystander T cells’ motility to support scanning of resident dendritic cells for better antigen recognition.

Although the field of purinergic receptors now contains quite a lot of substantial publications, the role of P2 receptors during immune system aging is still under investigation. Supportive data of a direct dysregulation on the receptors itself is missing. However, the evidence supports that alterations in the purinergic signaling pathways occur during aging. The survival of T_E_ cells, their specific cytotoxic competence, activity, and necessary transition into T_M_ cells is a critical step of the recall immune response strongly affected during aging. Additionally, there is evidence that changes in purinergic signaling pathways mediated by nucleotides influence inflammatory processes [230,231]. 

Two important enzymes in purinergic signaling are the ectoenzymes ectonucleoside triphosphate diphosphohydrolase-1 (NTPDase1, CD39) and ecto-5′-nucleotidase (CD73). They are expressed on endothelial and immune cells and play a central role in inflammation [232,233,234] and tumor immunity [235,236,237]. Severe P2 receptor-mediated stimulation of endothelium, lymphocytes, and monocytes might cause a pro-inflammatory environment [238]. The activation of the P2X7 receptor inhibits Tregs’ immunosuppressive potential and induces their conversion to Th17 effector cells in vivo during inflammation by increasing ATP levels via IL-6 [219,239,240]. The CD39/CD73 pathway counteracts through the degradation of excessive ATP levels into adenosine, leading to a more anti-inflammatory environment [241]. Interestingly Fang and colleagues identified CD39 as a cell surface marker for short-lived CD4^+^ effector T cells [242]. Furthermore, CD39 has been reported on exhausted CD8^+^ T cells [243] and CD8^+^ T_M_ cells are more prone to express CD39 than CD4^+^ T cells [244]. In mice, they may function as an integral component of T cells’ suppressive machinery, as Tregs express CD39 and CD73 [245] and impact Th17 cell generation [246]. Increased induction of CD39 with age on human CD4^+^ T cells correlates with increased apoptosis after antigen encounter [242] and reduced generation of long-lived T_M_ cells in vaccine response. In agreement with the in vitro observations, individuals with CD39 polymorphisms [247] show higher efficiency to vaccination [242]. Still, the follicular helper T (Tfh) cells and the survival as T_M_ cells are compromised, leading to vaccination’s inefficacy. Overall the increased CD39 expression with age resulting in reduced ATP concentration and preventing signaling through P2X receptors will lead to higher apoptosis susceptibility and preferential generation of short-lived effector T cells [242]. 

Opposite to CD4^+^ T cells, isolated CD8^+^ T cells are overall relatively resistant to extracellular ATP [228,248,249]. Mellouk and colleague recently investigated the ATP-sensitivity of CD8^+^ T cells (T_N_, T_CM/EM_) isolated from secondary lymphoid organs during aging [250]. They identified a CD44^hi^CD45RB^hi^ phenotype within the aged CD8^+^ T cell populations with total resistance to apoptosis induced by ATP in contrast to CD4^+^ T of the same age. Thus, their cytotoxic activity might be maintained even in inflammatory tissues where high ATP concentration is a common phenomenon [251], and the CD44^hi^CD45RB^lo^ phenotype will probably undergo apoptosis. The level of P2X7 receptor expression is upregulated on T_CM/EM_ CD44^hi^CD45RB^lo^ cells compared to T_N_, but low on CD44^hi^CD45RB^hi^ T cells and unaffected with aging. Furthermore, the P2X7R^lo^CD44^hi^CD45RB^hi^ phenotype with aging is entirely resistant to ATP-mediated channel formation and Ca^2+^ influx. The data suggest a minor role of the ATP/P2X7 receptor pathway in CD8^+^ T cell activation and differentiation during secondary immune responses [250].

## 8. Perspectives

**Figure 1:**
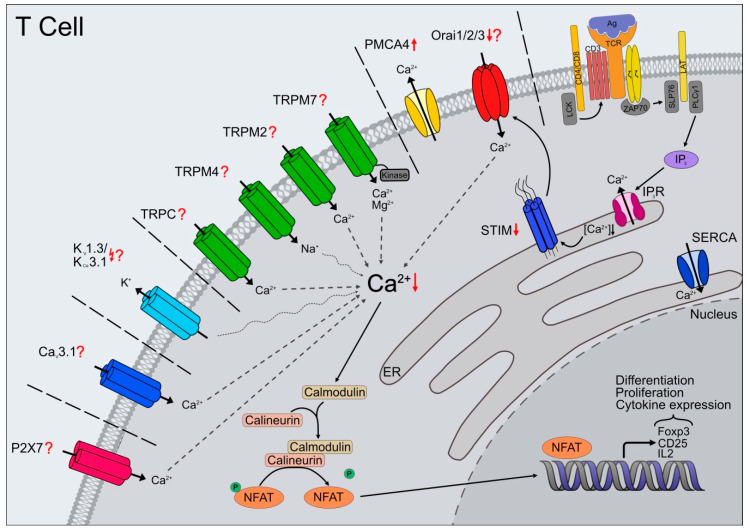
Altered Ca^2+^ signaling in T cells during aging—impact of ion channel expression and function. Calcium signaling in T cells, mediated by several types of channels, pumps, and receptors, is part of a crucial activation pathway, leading to proliferation, differentiation, and various effector functions. Antigen recognition through T cell receptors (TCRs) initiates the phosphorylation of multiple adaptor proteins. Activation of PLCγ1, followed by inositol triphosphate (IP_3_) production, induces Ca^2+^ release from ER calcium stores by binding to its receptor (IP_3_R). The decrease of [Ca^2+^]_ER_ activates STIM, which translocates to the plasma membrane and causes store-operated calcium entry (SOCE) through direct interactions with Orai channels. The increased cytosolic Ca^2+^ concentration leads to the activation of calcineurin, dephosphorylation and nuclear translocation of NFAT resulting in expression of IL2, CD25, Foxp3, and further components essential for T cell function. Other ion channels including (non-selective) TRPC and TRPM2/7, Ca_V_ channels (e.g., Ca_V_3.1), and purinergic ionotropic receptors (e.g., P2X7) mediate Ca^2+^ influx during T cell activation. Additionally, ion channels like potassium channels (e.g., K_V_1.3, K_Ca_3.1) or TRPM4 indirectly regulate Ca^2+^ influx through de- or hyperpolarization of the plasma membrane. Besides alterations in TCR activation, also Ca^2+^ signaling changes during aging. STIM1 and STIM2 expression levels are reduced (↓). Orai channel expression is still under investigation but Orai2 mRNA expression is decreased (↓). In contrast, PMCA4 expression increases with age (↑) leading to higher Ca^2+^ extrusion. Inhibition of K_V_1.3 or K_Ca_3.1 causes age- and subtype- dependent differences in Ca^2+^ influx patterns (↯). Possible changes in expression or function of other ion channels during aging are largely unknown (?).

Through increasing numbers of studies, it becomes more evident that ion signal transduction changes appear to have a strong influence on the development of age-associated diseases. Knowing the significant role of Ca^2+^ signaling for immune cells, a better understanding of ion channel and receptor biology is essential for the development of effective and targeted treatment strategies. Especially the ongoing SARS-CoV-2 pandemic reminds us how essential a functioning immune system is—the risk for severe illness with COVID-19 increases with age. The remodeled immune system of the elderly, with less naïve T cells, dysfunctional memory cells, and altered innate immune response, leads to greater susceptibility to infectious disease. Moreover, vaccinations do not always seem to provide sufficient immunity for the elderly, with less immunogenicity and effectiveness in the elderly than younger individuals [252]. Since immunological memory is the basis of vaccination, it is essential to understand the different T cell subsets’ changes during aging. T cells are extremely heterogeneous in terms of longevity, phenotype, distribution, and function, and the changes brought about by aging increase this complexity even further. Additionally, changes in the abundance and functionality of the Ca^2+^ and K^+^ channels may contribute to altered Ca^2+^ homeostasis in T cell subsets during aging (Figure 1). Therefore, more profound understanding of the dysregulation of ion channels contributing to the altered ion signaling transduction in immune cells with age is indispensable. More detailed and sophisticated studies are necessary to place channel and receptor dysfunction as a possible hallmark of aging.
